# Thyroid heterogeneity, as indicated by the CV of ultrasonographic intensities, correlates with anti-thyroid peroxidase antibodies in euthyroid Hashimoto’s thyroiditis

**DOI:** 10.1186/1756-6614-6-5

**Published:** 2013-03-23

**Authors:** Yosuke Wakita, Toshiki Nagasaki, Yuki Nagata, Yasuo Imanishi, Shinsuke Yamada, Koichiro Yoda, Masanori Emoto, Eiji Ishimura, Masaaki Inaba

**Affiliations:** 1 Department of Metabolism, Endocrinology and Molecular Medicine, Osaka City University Graduate School of Medicine, 1-4-3, Asahi-machi, Abeno-ku, Osaka-city 545-8585, Japan

**Keywords:** Hashimoto’s thyroiditis, Heterogeneity, Ultrasonography, Anti-thyroid peroxidase antibodies

## Abstract

**Objective:**

To prospectively evaluate the heterogeneous appearance of the thyroid gland, reflecting inflammation and destruction in euthyroid Hashimoto’s thyroiditis (HT), we investigated the clinical utilities of the heterogeneity index (HI) [the coefficient of variance (CV) of the ultrasonographic (US) intensities], focusing on anti-thyroid peroxidase antibodies (TPO-Ab), which represent not only disease activity but also subsequent thyroid destruction of HT.

**Methods:**

Forty-four consecutive patients with euthyroid HT [60.5 ± 2.7 years old (mean ± SE)] and 30 age-matched normal controls were studied. HI was calculated as the CV (SD/mean) of US intensities of either four points per lobe of the thyroid gland along a horizontal line at the depth of the right common carotid artery. Evaluation included serum levels of free thyroxine (FT4), free triiodothyronine (FT3), thyroid stimulating hormone (TSH), anti-thyroid peroxidase antibodies (TPO-Ab), anti-thyroglobulin antibodies (Tg-Ab), thyroglobulin and thyroid volume.

**Results:**

While no differences were observed for TSH, FT_4_ and FT_3,_ thyroglobulin and thyroid volume between the two groups, HI exhibited a tendency towards a significant difference (3.59 ± 0.20% in HT patients *vs* 3.23 ± 0.19% in normal group, p = 0.089). In HT patients, there was a significant and positive correlation of HI with TPO-Ab (r = 0.396, p = 0.034), whereas such a correlation was absent in normal controls. In both groups, there were no significant correlations of HI with Tg-Ab, FT_3_, FT_4_ or TSH.

**Conclusions:**

This is the first report of the close relation between heterogeneity of US of the thyroid gland and TPO-Ab in euthyroid HT patients before the heterogeneity becomes distinguishable from normal thyroid glands. Furthermore, at this stage, subsequent thyroid destruction in HT might be already be predicted through the heterogeniety of the thyroid tissue.

## Introduction

Ultrasonographic (US) examination is a very accurate and highly sensitive method for assessing thyroid gland lesions, especially thyroid nodules, that can be performed quickly even in out-patient clinics [1]. Nevertheless, few studies have reported the use of US to estimate inflammation and destruction of thyroid parenchymal tissue. While hypoechogenicity has been reported in untreated and levothyroxine-treated overt hypothyroid Hashimoto’s thyroiditis (HT) patients [2,3], the clinical utility of measuring US intensities remains unknown in euthyroid HT without apparent hypoechogenicity.

In the current report, we used a newly-designed sensitive US method that was able to distinguish from the hypoechogenicity [2,3] and enabled to assess and quantify heterogeneity in the euthyroid state, as the heterogeneity index (HI) [CV of the US intensities of eight points of thyroid gland on horizontal line between bilateral common carotid arteries]. This method was not affected by depth of the thyroid gland and weakening of ultrasound because it was based on the CV (SD/mean) of the US intensities along a horizontal line.

For patients with HT, a highly sensitive radioimmunoassay system for anti-thyroid antibodies [anti-thyroglobulin antibodies (Tg-Ab) and anti-thyroid peroxidase antibodies (TPO-Ab)] has proven to be essential for diagnosis [4]. In particular, TPO-Ab has been established to represent the current activity of HT and subsequent thyroid destruction, because the TPO-antigen is closely involved with cell-mediated cytotoxicity, whereas the Tg-Ab is not [5].

Therefore, we investigated the clinical meaning of HI in euthyroid HT patients, and attempted to correlate HI with a variety of thyroid markers, such as thyroid hormones, thyroglobulin and thyroid volume, and autoimmune anti-thyroid antibodies.

## Patients and methods

### Subjects

The study was approved by the ethical review committee of Osaka City University Hospital. Written informed consent was obtained from each patient. Newly detected 56 HT patients in a euthyroid state (M/F, 10/40) were enrolled consecutively during the 7-month period from January to July, 2011.

The diagnosis of HT was made based on the elevation of either Tg-Ab or TPO-Ab above the normal upper limit. To confirm sustained euthyroidism, thus excluding patients with a temporary condition, i.e., during the recovery from painless thyroiditis or occult subacute thyroiditis, measurement of TSH was conducted at least twice over 6 months. Moreover, to avoid confounding factors known to affect thyroid hormones, patients who were pregnant or post-partum [6], patients receiving other hormone-replacement therapy or taking any drugs that could affect thyroid hormones, such as amiodarone hydrochloride, interferon and Interleukin-2, lithium carbonate, gonadotropin releasing hormone, raloxifene hydrochloride, anti-convulsion drugs, and rifampicin, and those with autoimmune antibodies, were excluded from the study [7,8].

Normal control subjects who joined the Health-Check Program at Osaka City University Hospital were enrolled consecutively as age-matched controls (M/F, 10/40). Normal controls had no history of thyroid disease, had neither goiter nor an autoimmune-antibody titer, and were in a euthyroid state.

The smoking index (the daily number of cigarettes multiplied by the number of years of smoking) was similar between the HT patients and normal controls and individual smoking habits did not change during this study.

#### Serum parameters

Blood was drawn just before ultrasonography after overnight fasting. Commercially available electrochemiluminescent immunoassay (ECLIA) kits were used for the thyroid hormone assay. TSH was measured by sandwich Elecsys^®^, and FT4 and FT3 levels were determined by competitive Elecsys^®^ (Roche Diagnostics K.K., Tokyo, Japan) [9]. Anti-thyroglobulin (Cosmic Co., Tokyo, Japan) and anti-thyroid peroxidase antibodies (Cosmic Co., Tokyo, Japan) were also determined using highly-sensitive, commercially available, radioimmunoassay kits [10]. In this system, highly purified thyroglobulin and thyroid peroxidase were used as antigens. Serum concentrations of anti-thyroglobulin and anti-thyroid peroxidase antibodies greater than 0.4 and 0.3 U/ml, respectively, were considered positive. Serum thyroglobulin levels were measured using an ECLIA kit, Elecsys^®^ (Roche Diagnostics K.K., Tokyo, Japan) [11].

#### Quantitative ultrasonographic measurements of heterogeneity index and thyroid volume

**Figure 1:**
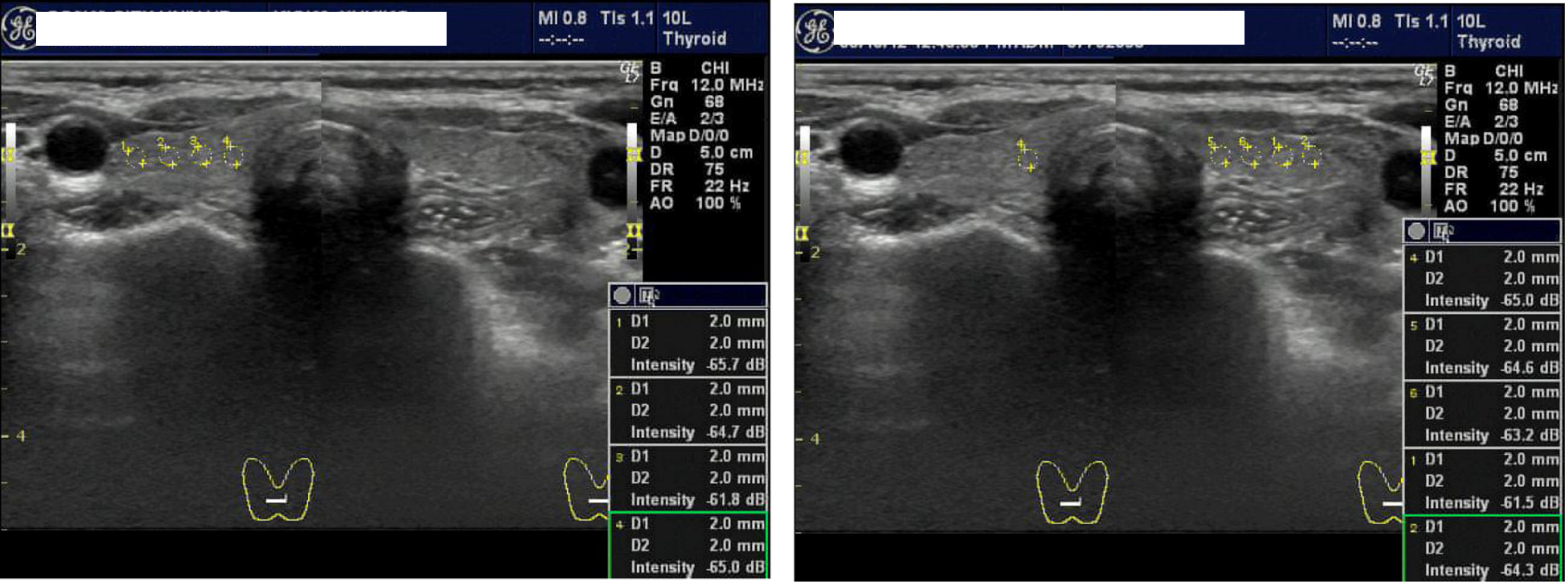
**Recording of the ultrasonographic intensities and calculation of the thyroid heterogeneity index (HI).** The appropriate sampling points were first determined for measuring. Four open-circle cursors (2 mm in diameter) per half lobe were positioned straight along a horizontal line at the depth of the right common carotid artery adjacent to the thyroid gland. US intensity within each circle was automatically calculated immediately after the cursor was set. HI was defined as the CV (i.e., SD/mean) of eight US intensities.

Thyroid HI was determined using an US apparatus (LOGIQ 7PRO, GE healthcare, Princeton, NJ, USA) with a 10-MHz linear array probe operating in B-mode (Figure 1). Four open-circle cursors (2 mm in diameter) per half lobe were positioned along a horizontal line at the depth of the right common carotid artery adjacent to the thyroid gland. US intensity within each circle was automatically calculated immediately after one cursor was set. HI was defined as the CV (SD/mean) of eight US intensities. All measurements were performed by the same examiner (Y.N.), who was blinded to the characteristics of the subjects.

Thyroid volume was also determined by ultrasonography, based on a calculation using an ellipsoid model (width × length × thickness × 0.7 for each lobe) [12].

#### Statistical analysis

Data are expressed as the mean ± SE unless otherwise indicated. Statistical analyses were performed using StatView (version 5.0, SAS Institute, Cary, NC). Differences in clinical factors between HT patients and normal controls were examined using the Mann–Whitney *U* test for assessment of the median values. The difference in the male/female ratio between the two groups was analyzed using the chi-square test. Simple regression analysis was used to examine the relationships between the two factors. *P*-values less than 0.05 were considered to be statistically significant.

## Results

### Clinical variables in normal controls and HT patients

A comparison of the variables between normal controls and HT patients is presented in Table 1. In addition to common biochemical markers, such as liver enzymes, renal function and lipid profiles, no significant differences were found in age, male/female ratio, BMI, smoking index, the serum level of TSH, FT_3_ and FT_4_, thyroglobulin, or thyroid volume between normal controls and HT patients (data not shown).

**Table 1:** Baseline characteristics of the subjects with Hashimoto’s thyroiditis and the controls

	**Subjects with Hashimoto’s thyroiditis**	**Normal controls**	**P**
Number of subjects	44	44	
Gender (female/male)	32/12	32/12	ns
Age (years)	60.5 ± 2.7	58.7 ± 1.9	ns
Body mass index (kg/m^2^)	21.7 ± 0.48	21.4 ± 0.58	ns
Smoking index	74 ± 59	67 ± 72	ns
Tg-Ab (U/mI) [<0.4]	8.37 ± 2.55	<0.4	
TPO-Ab (U/mI) [<0.3]	15.3 ± 6.13	<0.3	
FT_4_ (pmol/l) [11.6-21.9]	14.5 ± 0.37	14.6 ± 0.57	ns
FT_3_ (pmol/l) [3.54-6.16]	4.17 ± 0.11	3.95 ± 0.15	ns
TSH (mIU/l) [0.5-5]	2.40 ± 0.33	2.53 ± 0.28	ns
Thyroglobulin (mg/l) [0.0-30.0]	43.4 ± 20.6	40.6 ± 11.5	ns
Thyroid volume (mm^3^)	14687.1 ± 1494.3	15757.2 ± 1984.4	ns
HI (%)	3.23 ± 0.19	3.59 ± 0.20	ns(0.089)

Data are expressed as the mean ± SE. The differences between two groups were examined using the Mann–Whitney *U* test for assessment of the medians.

*ns*: not significant, *Systolic BP*: systolic blood pressure, *Diastolic BP*: diastolic blood pressure, *Tg-Ab*; anti-thyroglobulin antibody, *TPO-Ab*; antithyroid peroxidase antibody, *HI*: heterogeneity index.

### HI in normal controls and HT patients

As shown in Table 1, HI exhibited a tendency towards a significant difference between normal controls and HT patients (3.23 ± 0.19% in the normal group vs. 3.59 ± 0.20% in the HT patients, p = 0.089).

### Correlations between HI and clinical variables in normal controls and HT patients

The correlations between HI and the clinical variables in normal controls and HT patients are presented in Table 2. In the HT patients there was a significant and positive correlation between HI and TPO-Ab (r = 0.396, p = 0.034) but not Tg-Ab (r = −0.014, p = 0.941) or TSH (r = −0.012, p = 0.953). In normal controls HI did not correlate with these factors. There were no significant correlations between HI and age, BMI, smoking index, serum level of FT_3_, FT_4_, thyroglobulin or thyroid volume in either group (data not shown).

**Table 2:** Correlations between the heterogeneity index and other parameters in subjects with Hashimoto’s thyroiditis and controls

	**Subjects with Hashimoto’s thyroiditis**		**Normal controls**	
	**r**	**P**	**r**	**P**
Age (years)	0.077	ns	0.082	ns
Body mass index	0.253	ns	0.201	ns
Smoking index	0.292	ns	0.201	ns
Systolic BP	0.044	ns	0.153	ns
Diastolic BP	−0.045	ns	0.279	ns
Pulse rate	−0.143	ns	−0.017	ns
Tg-Ab	−0.014	ns		
TPO-Ab	0.396	0.034		
FT_4_	−0.045	ns	0.188	ns
FT_3_	−0.239	ns	−0.048	ns
TSH	−0.125	ns	−0.172	ns
Thyroglobulin	−0175	ns	−0.204	ns
Thyroid volume	0.264	ns	0. 175	ns

Data are expressed as the mean ± SE. Simple regression analysis was used.

ns: not significant, Systolic *BP*: systolic blood pressure, Diastolic *BP*: diastolic blood pressure, *Tg-Ab*; anti-thyroglobulin antibody, *TPO-Ab*; antithyroid peroxidase antibody, *HI*: heterogeneity index.

**Figure 2:**
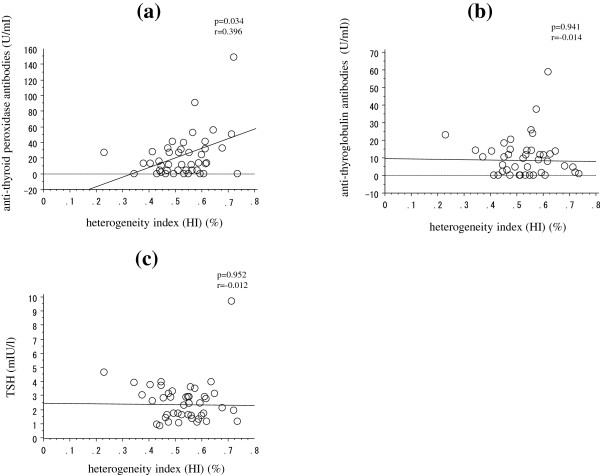
**Simple regression analysis to determine the correlation of the heterogeneity index (HI) with anti-thyroid peroxidase antibody (TPO-Ab) (a), anti-thyroglobulin antibody (Tg-Ab) (b), and TSH (c) in Hashimoto’s thyroiditis (HT).** HI correlated significantly with TPO-Ab (r = 0.396, p = 0.034), but not with HI and Tg-Ab or TSH (r = −0.014, p = 0.941 and r = −0.012, p = 0.953, respectively).

Figure 2 presents the correlations between HI and TPO-Ab, Tg-Ab and TSH in the HT patients.

## Discussion

The results of the study revealed a significant and positive correlation between HI and TPO-Ab in euthyroid HT patients. In these patients, HI might reflect not only the extent of current inflammation and destruction of the thyroid tissue, but also subsequent destruction in HT, because TPO-Ab is responsible for these changes through an autoimmune reaction in HT [5]. However, in the cases with very mild destruction, this correlation likely suggests the subsequent potential for terminal destruction.

TPO, originally described as thyroid microsomal antigen, is present on the apical surface of thyroid follicular cells and is the antigen most closely involved in cell-mediated cytotoxicity [13]. Hence, among auto-immune antibodies specific for the thyroid gland, the TPO-Ab titer represents the degree of lymphocytic infiltration of the thyroid gland, reflecting the current activity of HT, as well as subsequent stages in the development of HT [5,13].

Nevertheless, since only a tendency of HI was found between HT patients and normal controls (Table 1), inflammation and destruction of the thyroid tissue would likely be slight or mild in the early phase of HT, namely the euthyroid state. Therefore, the clinical interpretation of HI might be not the degree of current inflammation and destruction but more likely a measure of the potential for terminal destruction of the thyroid gland that can be achieved in the early stages of HT.

In a previous report, an interaction between the HLA-DRB4 and cytotoxic T-lymphocyte associated antigen 4 (CTLA-4) genes was shown to determine the thyroid function of TPO-positive Japanese HT patients with goiter [13]. This is consistent with our findings, supporting that terminal destruction of thyroid gland is genetically predisposed.

Meanwhile, Tg-Ab, another auto-immune anti-thyroid antibody [4], did not correlate with HI in HT patients (Figure 2). Multiple antigen configurations of thyroglobulin are produced when it becomes iodinated, resulting in functionally active but immunologically distinct molecules [13]. Therefore, changes in Tg-Ab could occur independent of immune responses or the consequent inflammation and destruction in HT.

The lack of correlation of HI with FT_3_, FT_4_ and TSH in HT patients (Table 2) might be explained by similar degrees of heterogeneity and thyroid hormones between HT patients and normal controls. Thus, in the euthyroid state of early HT inflammation, the simultaneous destruction of thyroid tissue had not progressed sufficiently to affect these factors. If measured in a stage of overt hypothyroidism, a correlation might be observed between HI and thyroid hormones, as was reported previously, with respect to hypoechogenicity in HT [2,3]. This is the subject of ongoing and future investigations by our group.

Serum thyroglobulin did not correlate with HI in this study (Table 2). Possible explanations for this are as follows: although thyroglobulin is intimately associated with the thyroid, it is a normal component of the blood, as some thyroglobulin is invariably secreted, during normal thyroid hormone release [14]. Therefore, the elevation of serum thyroglobulin in HT could be due to increased leak in response to TSH stimulation or the excessive production by the associated cancer. Moreover, problems include variable sensitivity and reproducibility of the assays and interference by thyroglobulin autoantibodies [15]. Hence, in the euthyroid state the existence of Tg-Ab thyroglobulin may be of less clinical importance.

HI did not correlate with thyroid volume (Table 2), likely due to the same reason of the early stage of HT; i.e., in the presence of a very mild inflammation and early destruction of thyroid tissue in the euthyroid state of HT, the changes in thyroid volume did not reach statistical significance. In another previous investigation of overt autoimmune hypothyroidism, thyroid volume correlated negatively with echogenicity [16].

HI is easily and reproducibly obtained using ultrasonography, and is not affected by the depth of the thyroid gland and weakening of ultrasound, because it represents the CV (SD/mean) of US intensities along a horizontal line.

Thus, computerized grey-scale analysis using US was previously reported to be useful in quantifying thyroid hypoechogenicity in untreated and levothyroxine-treated overt hypothyroid HT patients [7]. The marked difference from our method is not in the assessment of heterogeneity but in the quantification of hypoechogenicity, which would be affected by the depth of the thyroid gland and weakening of the ultrasound. Furthermore, while their subjects included untreated and levothyroxine-treated overt hypothyroid HT patients, our method was applied to euthyroid and subclinical hypothyroid patients with visually indistinguishable heterogeneity.

Further, computerized grey-scale analysis allows for significant correlations between hypoechogenicity and indices of the thyroid gland, namely serum TSH, FT4 and TPO-Ab values. Interestingly, even in overt hypothyroidism echogenicity did not correlate with Tg-Ab, but rather correlated with TPO-Ab, possibly due to the same underlying reasons noted above.

One limitation of HI is obstruction by the tumor or cyst along the horizontal line for sampling, which interferes with positioning of the four open-circle cursors for each half lobe. Another limitation occurs when the thyroid gland is too small or atrophic, and cursors cannot be positioned properly. Since each open-circle is 2 mm in diameter, a straight line more than 8 mm in length is needed for each lobe.

In summary, our results demonstrate the close relation between heterogeneity of the thyroid gland and TPO-Ab in euthyroid and subclinical hypothyroid patients with Hashimoto’s thyroiditis. It was suggested that even in such hormonal states, without distinguishable inflammation or destruction of thyroid tissue compared with normal subjects, subsequent thyroid destruction of HT might already be indicated through heterogeniety of the thyroid tissue.

## Competing interests

We declare that there is no competing interests that could be perceived as prejudicing the impartiality of the research reported.

## Authors’ contributions

YW carried out the ultrasonographic measurements and analyzed the data. TN drafted the protocol and manuscript, analyzed the data. YN assisted ultrasonographic measurements and analysis of the data. YI, SY, KY, ME, EI and MI participated in the logical composition of manuscript. All authors read and approved the final manuscript.
